# Estimating time-to-contact when vision is impaired

**DOI:** 10.1038/s41598-021-00331-5

**Published:** 2021-10-27

**Authors:** Heiko Hecht, Esther Brendel, Marlene Wessels, Christoph Bernhard

**Affiliations:** Psychologisches Institut, Johannes Gutenberg-Universität Mainz, Abteilung Allgemeine Experimentelle Psychologie, Wallstraße 3, 55099 Mainz, Germany

**Keywords:** Psychology, Human behaviour

## Abstract

Often, we have to rely on limited information when judging time-to-contact (TTC), as for example, when driving in foul weather, or in situations where we would need reading glasses but do not have them handy. However, most existing studies on the ability to judge TTC have worked with optimal visual stimuli. In a prediction motion task, we explored to what extent TTC estimation is affected by visual stimulus degradation. A simple computer-simulated object approached the observer at constant speed either with clear or impaired vision. It was occluded after 1 or 1.5 s. The observers extrapolated the object’s motion and pressed a button when they thought the object would have collided with them. We found that dioptric blur and simulated snowfall shortened TTC-estimates. Contrast reduction produced by a virtual semi-transparent mask lengthened TTC estimates, which could be the result of distance overestimation or speed underestimation induced by the lower contrast or the increased luminance of the mask. We additionally explored the potential influence of arousal and valence, although they played a minor role for basic TTC estimation. Our findings suggest that vision impairments have adverse effects on TTC estimation, depending on the specific type of degradation and the changes of the visual environmental cues which they cause.

## Introduction

Since Lee^1^ proposed that observers perform time-to-contact (TTC) estimates of an approaching object, without having to rely on information about the object’s distance or size, a great number of studies have elaborated on the variables necessary and/or sufficient to perform TTC estimates^2–4^. Normal adequate viewing conditions have been studied abundantly, and performance tends to be rather good. We can successfully catch an approaching ball^5,6^ or safely cross a busy road. We may do so by using a critical TTC-value to initiate actions. For instance, in a driving simulator study, it was found that drivers initiate braking at a constant TTC to a decelerating preceding vehicle^7^. In contrast, we know little about TTC estimation when viewing conditions are poor or the visual stimulus is degraded. Stimulus degradations can be induced by two different venues. Firstly, the stimulus can be obscured by changes in lighting, fog, rain, and other external environmental factors. Secondly, the perception of the stimulus can be compromised internally by the state of the observer, be it for systemic (fatigue, inebriation) or pathological reasons (cataracts, reduced visual acuity, etc.). Here, we report two experiments we conducted to investigate TTC estimation under different conditions of visual degradation.

Poor visibility affects visuo-motor performance, but to what extent are TTC estimates affected? For instance, precipitation is associated with large increases in traffic collisions and injuries^8^, and driving with impaired vision is associated with an increased risk of at-fault crashes^9^. Besides the obvious effects of precipitation and impaired vision—like a slippery ground or not detecting an obstacle in time—more subtle effects of such conditions on visual perception may also be at work. Among the latter, we chose to examine the role of complex visual impairment on TTC estimation, as brought about by fog, poor eye-sight, or snowfall. Note that as a consequence, the stimulus manipulations were not always pure, for instance reduced contrast also increased luminance. Such contrast reduction can ensue as a consequence of changes in ambient lighting or when suffering from cataracts. Dioptric blur arises when observers are near-sighted or far-sighted without receiving corrective lenses. And snowfall obscures the entire scene. All of these, and other stimulus degradations may trigger an emotional response, as well as a safety-based response strategy if the degradation is deemed potentially dangerous. To be able to gauge the importance of emotional aspects, we have additionally varied the emotional content of the approaching stimuli. Thus, we chose to create conditions of visual degradation that could have their cause in the environment or in the observer and used stimuli of different emotional content. Moreover, to disentangle the basic effect of vision degradation on TTC estimation from driving-specific effects, we decided to test TTC estimation in a simple task unrelated to driving, although previous studies have often investigated visual impairment and potential coping strategies in the context of driving. The effects of vision degradation on perception and performance will be outlined in the following section.

### Types of stimulus degradation

#### Contrast reduction and dioptric blur

Both internal observer-related and external environment-related factors can cause contrast reduction or blur. Cataracts and fog are examples for such degradation. Among the most common symptoms of cataracts are cloudy or blurry vision and the phenomenon that colors seem faded^10^. Such reductions of contrast and/or contrast sensitivity are associated with self-reported driving difficulty^11,12^, increased at-fault crash risk^9,13^ as well as performance changes.

As outlined, impaired vision is particularly relevant in driving contexts, and many studies investigating the role of reduced contrast adopted this context. Despite this contextual similarity, some studies reported mixed results. For example, reducing contrast in a video-based driving simulator made speeds not only appear slower but also harder to discriminate^12^. However, a more sophisticated driving simulation study using 2D and 3D stimuli found maintenance of precision with reduced contrast. That is, the ability for speed discrimination remained unaffected in the case of low contrast as accomplished by compressing gamut range but maintaining overall luminance^14^, but the study confirmed that in low contrast conditions, speed perception suffered in accuracy. Aside from the effect of contrast, other experiments have observed changes in luminance to affect speed perception, in terms of speed underestimation with increased luminance^15–17^.

Even if the degradation due to uniform contrast differs from that due to fog, studies implementing foggy scenes in a driving simulator also reported a diminished sense of speed. That is, objects were perceived to be moving more slowly^18^ and drivers showed greater speed matching errors in a car-following task^19^. As outlined by Pretto et al.^20^, these effects of reduced contrast and fog on speed perception seem to depend on the altered spatial distribution of contrast. When contrast was reduced uniformly or increased with distance, speed was underestimated. However, when contrast decreased with distance, as in real fog, speed was overestimated. Additionally, contrast reduction might also affect the perception of distance. Previous experiments observed an overestimation of egocentric distance in fog^21,22^, presumably due to aerial perspective^23^. Dense fog obscuring the outline of a leading vehicle even led to longer reaction times when judging the distance to a lead car^10^.

Note, however, that an assessment of velocity and/or distance may or may not be used for TTC estimation^1,24–26^. The use of optical variables has been demonstrated when observers had to catch a ball of known size^27^. They relied on the object’s changing visual angle, or its expansion rate when the approaching object was the periphery^28^. In other cases, for instance when confronted with centrally approaching objects, simpler visual cues often guide perception. The fact that observers appear flexible at adopting one or another strategy has often caused contention, especially in driving contexts^26^. We hold the view that the visual system uses an adaptive strategy, using a simple cue only if it suffices to guide action, which is often distance or velocity-based^24,29–31^. For example, in a real driving task, contrast reduction—when noticed—resulted in slower speed traveled, but neither consistently altered verbal estimates of speed nor stopping distances^32^. This speaks for a distinguishable effect of contrast reduction on the control and perception of speed. However, it also suggests that contrast reduction does not necessarily affect TTC estimation, as an indicator of estimated stopping distance, even if it affected speed control behavior. On the other hand, there is evidence for a direct effect of reduced contrast on collision detection sensitivity in older drivers ^33^, which presumably relies on similar mechanisms as TTC estimation.

Regarding TTC estimation in a non-driving context, we examined the direct effect of contrast in a previous experiment^34^. We investigated looming objects of changing contrast with respect to the background, as would be common when a cloud obscures parts of the scene. However, no significant effect of contrast variation on TTC estimation was observed. Battaglini et al.^35^ additionally examined the effect of reduced contrast of simple objects on TTC estimation in a horizontal approach task and observed that contrast reductions produced longer estimated TTC. In the current study, we also implement a basic, non-driving context but focus on a different kind of contrast reduction, where reduced contrast accompanied by higher luminance decreases the visibility of the entire scene.

The role of image blur or defocused images is even less clear. A visual degradation as induced by blur changes the spatial frequency as it attenuates high spatial frequencies, which is comparable to a low-pass filter. For low-pass filtered objects, there is evidence that they appear slower than unfiltered objects^36^. However, research has either found a weak or no association between visual acuity impairment and collision involvement rates, even if these impairments resulted in inferior driving performance^13^. Its effect on TTC estimation had not yet been explored. In Experiment 1, we therefore also examine the basic perceptual effect of blur on visual TTC estimation.

#### Snowfall

Another source that could affect TTC estimation is an external stimulus degradation that not only affects the visibility of the object but also introduces unrelated distracting motion into the scene, as is the case in heavy rain or snowfall. In a trend-analysis of Canadian weather and collision data between 1984 and 2002, Andrey^37^ found that rainfall increased the risk of fatal car accidents. Lately, this risk increase has dropped from 90 to 50% above normal driving conditions, possibly due to advanced driving assistance systems, such as anti-lock braking systems. In contrast, the relative risk of casualty during snowfall stayed at constant 87% above normal. The more prominent distracting motion signals introduced by snowfall could therefore affect perception on a more basic level that is difficult to counteract with technical aids. We decided to investigate the effect of distracting surround motion signals on TTC estimation by simulating snowfall.

But by which mechanism could surrounding motion signals affect TTC estimation? The detection of looming objects is based on two separate mechanisms, one for optic flow and another for scale changes. Neither of these two mechanisms is affected by adaptation of the other^38^. The optic flow processing, in particular, might be encumbered by distracting motion of the entire visual scene as introduced by snow. For instance, task-irrelevant local texture motion affected TTC estimation^39,40^, whereas a motionless textured background had little effect^41^. In a similar fashion, global motion patterns of random dot kinematograms caused interference with TTC estimation^42,43^. And the optic flow in the surrounding visual field, which signals observer motion, can also influence the TTC estimates of an independently approaching object. Estimated TTC of an approaching object decreases with forward self-motion^5,44,45^ and increases with backward self-motion^44^. The coherence of the optic flow appears to play an important role in the sense that it can be disregarded when incoherent but not when coherent^46^.

Falling snowflakes provide motion signals that constitute more or less coherent optic flow, which may signal self-motion if it becomes attached to the visual world, but not when regarded as noise. Thus, the increased motion signals introduced by snow could affect TTC estimation for approaching objects. They can be thought of as a special case of stimulus degradation. In Experiment 2, we investigate the effect of this motion signal.

## Experiment 1: contrast reduction and blur

The experiment described in this section was part of the second author’s academic thesis^47^. In this experiment, we investigated what effects visual impairments might have on frontal TTC estimation using a prediction motion paradigm. We introduced impairments by adding a semi-transparent mask to the entire stimulus, which reduced contrast and at the same time produced higher luminance, by blurring vision, or by a combination of both. Based on the above-mentioned findings, we hypothesized that reduced contrast accompanied by higher luminance would reduce perceived velocity and/or enlarge perceived distance, thus making TTC estimates longer (constant error). In contrast, blurred vision does not allow for a comparable prediction. Blur does not impede the global object-background separation per se, as does a uniformly reduced contrast, but blur reduces the sharpness of an object’s contours. On the one hand, blurred contours slightly enlarge the retinal image of an approaching object, which in turn might result in a larger perceived object size and consequently shorter TTC estimates, provided observers use a size heuristic. Moreover, the uncertainty of the stimulus outline might prompt a criterion shift toward more cautious behavior, suggesting shorter TTC estimates. On the other hand, the aforementioned finding of slower perceived speed for low-pass filtered objects^36^ suggests that blurred objects should also appear to move more slowly than crisp objects. Assuming that observers base their TTC estimation on perceived speed, TTC for blurred objects should be longer.

Note that regardless of whether perceived size, perceived speed, or some other cue guides TTC estimates, we hypothesized that reduced contrast and blur both constitute impairments that should increase the variation of the TTC estimations (variable error).

In a driving context, visual impairment poses a serious risk for safety as outlined in the introduction. Therefore, visual impairment could be accompanied by an emotional reaction of the driver, such as anxiety, and likewise higher levels of arousal. Emotional valence and arousal are interleaved and can affect TTC estimation^33–35^. For example, TTC for approaching threating objects was judged to be shorter than for neutral ones^48^. In a meaningful context, such as driving, the effects of contrast reduction and blur on TTC estimation could deviate from our expectations due to a moderating influence of arousal and/or valence. Thus, a possible influence on the TTC estimates measured in the prediction motion task seemed plausible. The estimates in such a task depend mainly on cognitive motion extrapolation^49^ and might thus be sensitive to cognitive modulations as caused by emotional processes. Consequently, we varied the valence and arousal of our stimuli together with the visual degradations. By doing so, we were able to differentiate the degradation effects from potential effects of valence and arousal on basic TTC estimation, and to explore whether valence and/or arousal moderate the relationship between visual degradations and TTC estimation.

### Method

#### Participants

24 observers volunteered to participate in Experiment 1, some for partial course credit (13 women, 11 men; age: 19–36 years, *M* = 24.15 years, *SD* = 4.53 years). Most of them were students at the University of Mainz, and all had normal or corrected-to-normal visual acuity and contrast sensitivity. The experimental procedures were in accordance with the principles of the Declaration of Helsinki^50^. The local ethics committee of Mainz University deemed approval of this experiment unnecessary. It issues a checklist of criteria that make approval mandatory. Among these are: participants need particular protection, the experimenter (initially) deceives participants about the purpose of the study, particularly large and comprehensive data sets are collected, the procedure may produce psychological stress or anxiety, the experiment involves brain imaging. None of the criteria apply to the work reported here. Informed consent was obtained from each participant.

#### Apparatus and stimuli

Displays were presented on a projection screen (2.5 m wide × 1.5 m high, Optoma HD20 projector, resolution 1920 × 1080 pixel) at 24 frames/s in a darkened room. The stimuli were rendered with a PC (Dell Precision 390; Intel Core 2 CPU-6400 2,13 GHz 4 GB RAM Windows 7 64 Bit, graphics card NVIDIA GeForce GTX 690). A chin rest stabilized head position at 2 m viewing distance. The stimuli were programmed in virtual space (Vizard 3.0); one virtual m corresponded to one real m.

**Figure 1:**
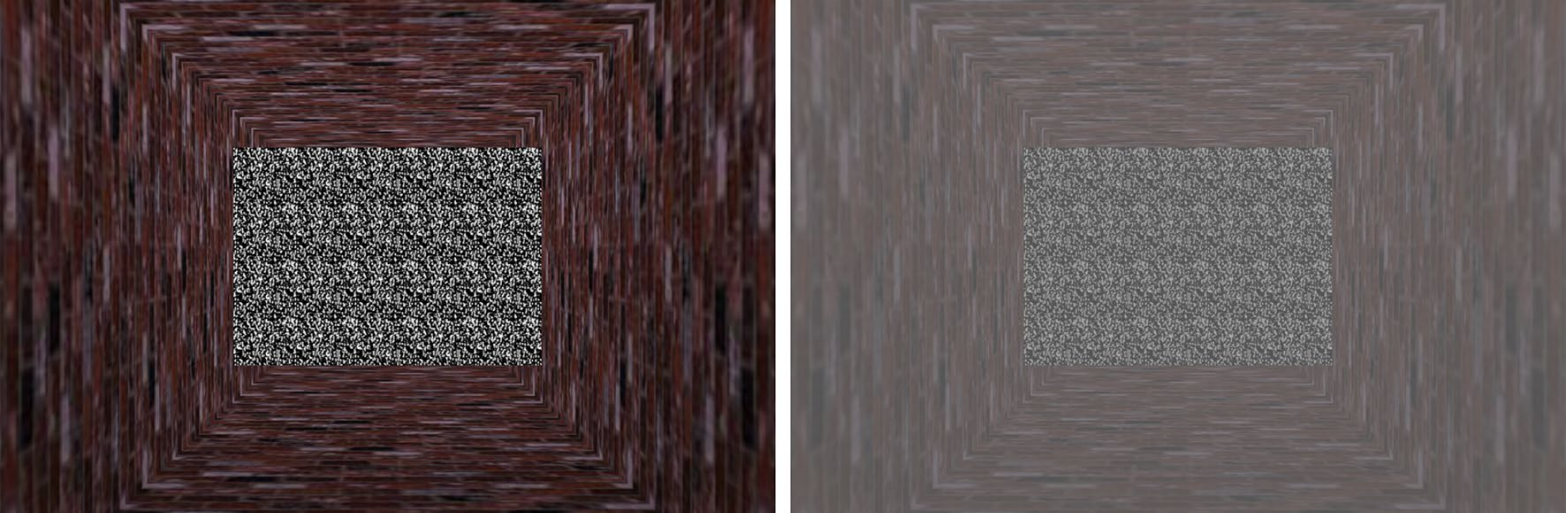
Example stimuli from the noise condition, with full contrast (left) and reduced contrast (right). Contrast reduction corresponded to a transparency of 15%.

Nine images from the International Affective Picture System (IAPS^51^) served as stimuli. They were mapped onto a flat object that appeared to approach the observer on a head-on collision course. The images represented three affective categories and were chosen from images we had used in earlier TTC experiments^52,53^. Three attack images consisted of a snarling Pit Bull, a masked attacker with a knife, and a biting snake, associated with high arousal and low valence ratings. We chose three erotic images with high arousal and high valence ratings, since they also elicit high levels of arousal but represent a counterpart to the attack images regarding valence. Finally, we chose three images for the neutral category, a man with a hat, a lamp, and plants, all associated with low arousal and intermediate valence ratings. The IAPS numbers of the pictures used were: 1120, 1300, 2190, 4311, 4659, 4695, 5000, 6510, 7175. As control condition, we created three noise images consisting of colored black-white, black-red and black-blue dots respectively. Figure 1 depicts two example stimuli from the noise condition with full and reduced contrast. As blur was manipulated by means of optician’s spectacles, blurred stimuli could not be depicted.

##### Simulated visual impairments

Participants wore an optometrist’s trial frame, into which lenses with different filters and of different dioptric values could be inserted. We used lenses with + 1.5 and + 3 diopters, which resulted in a blur of vision. Those lenses are equivalent to an uncorrected myopia of 1 and 2.5 diopters, respectively (since 0.5 diopters of accommodation are needed for clear vision at the viewing distance of 2 m). Lenses with 0 diopters served as control condition. In the *reduced contrast* condition, we used the Vizard software to place a virtual semi-transparent mask (RGB = [216, 216, 216]) with 15% transparency in front of the stimulus, which made the virtual environment appear as if seen through evenly fogged-up glasses. Unfortunately, contrast measures were not taken during data collection. A post-hoc analysis for a different monitor (BenQ PD3200Q) revealed a slight increase of overall luminance by approx. 32 cd/m^2^ for the black-white noise-target. The contrast changed from 21.6 to 1.4% in Michelson contrast. Note, however, that these differences were likely reduced for the projector used in the experiment and therefore represent a conservative control. We had previously used a similar reduction of contrast and blur successfully to simulate visual impairment^54^. For the purposes of the current study, we ascertained that the degradations did indeed reduce visual acuity and contrast sensitivity. The former was measured using the Landolt C set of the Freiburg Acuity Test (FrACT^55^). Contrast sensitivity was measured using a custom test based on the Pelli-Robson chart^56^. We conducted both tests prior to the experiment at 4 m viewing distance, which required an accommodation of about 0.25 diopters and avoided to run into the limits of display resolution. The added dioptric blur caused a marked and almost linear reduction in visual acuity and contrast sensitivity. The semi-transparent mask further reduced contrast sensitivity in all three dioptric blur conditions and visual acuity at 0 and -1.5 dioptric blur (for details see^54^, pp. 2401–2402).

#### Design and procedure

In a prediction motion paradigm a virtual flat object (2 m width × 1.5 m height) appeared at an initial distance between 11 and 7 m and disappeared at a final distance between 6 and 3.2 m from the observer. The respective visual angles ranged between 10.4° and 15.8° (initial) and between 18.9° and 34.7° (final). The object was simulated to approach the participant through a tunnel at constant velocity for 1 s, and then was blanked out, that is, it disappeared instantaneously. The tunnel, as if looking into the opening of a horizontal square brick chimney (see Fig. 1), remained visible. Participants had to extrapolate the motion of the object after it disappeared and to press a button when it would have collided with them (prediction motion paradigm). TTC estimates were calculated as the time from the object’s disappearance to the participant’s button press. To discourage participants from basing their judgments on simple heuristics, we varied approach velocity and actual TTC (time from disappearance to collision), which in turn determined initial starting distances. We chose TTC values below 1.5 s as this range is relevant in many motor actions and traffic encounters^57^.

The within-subjects repeated-measures design consisted of five fully crossed factors: *picture category* (4 levels: attack, erotic, neutral, noise), *velocity* (2 levels: 4 and 5 m/s), *TTC* (3 levels: 0.8, 1.0, and 1.2 s), *contrast* (2 levels: 100% transparency and 15% transparency), and *diopter value* (3 levels: 0, + 1.5, and + 3.0 diopters). Note that low contrast always went along with higher target luminance. Altogether, participants viewed 432 trials (12 pictures × 2 velocities × 3 TTCs × 2 contrast levels × 3 diopter values), starting each trial at their own pace. The three diopter-values and the two contrast levels were blocked; the order of the resulting six blocks was counterbalanced according to the method of latin squares. The order of the trials within each block was randomized. Six training trials with feedback (estimation error in ms and whether the button press occurred too early or too late) preceded the experiment, each without contrast reduction or dioptric blur, and with a photograph that was not used as an experimental stimulus.

At the end of the experiment, we assessed arousal and valence ratings for the stimuli in the same manner as these values were assessed for the IAPS catalogue. We showed each picture again for 6 s, asking the participants: “How do you feel when you look at this picture?”. The ratings were collected with self-assessment-manikins (SAMs^58^), a scale consisting of five sketches depicting the affective states. Participants can choose one of the manikins or an intermediate state, resulting in a 9-point scale.

### Results

#### Constant error

**Figure 2:**
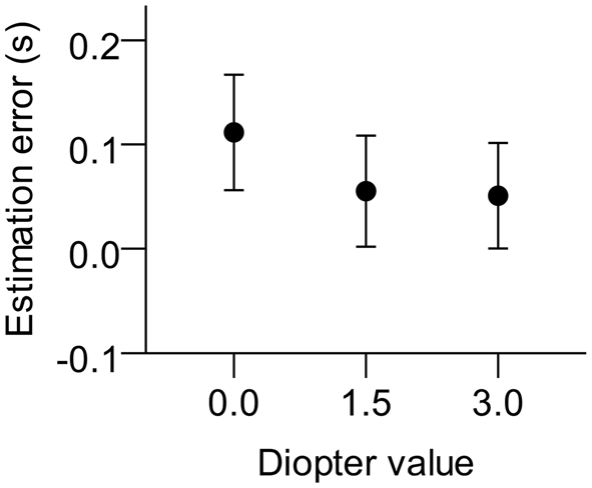
Mean TTC estimation errors (estimated TTC—actual TTC) as a function of diopter value. Error bars represent ± 1 standard error of the mean across participants.

We computed TTC estimation errors (estimated TTC – actual TTC), averaged them across the three trials per factor-combination, and analyzed those averages in a 4 (picture category) × 2 (velocity) × 3 (TTC) × 2 (contrast) × 3 (diopter value) ANOVA with repeated measures on all factors. For all data analyses described in this paper we used SPSS 21. Where appropriate, degrees of freedom reflect Huynh–Feldt corrections^59^. Figure 2 shows mean constant errors averaged per blur condition. Corresponding significant type 3 tests can be found in Table 1.

**Table 1:** Significant main and interaction effects of type 3 *F*-tests on mean constant errors.

	*df*_*Num*_	*df*_*Den*_	*F*	*p*	η_*p*_^*2*^
Picture category	2.36	54.33	6.88	.001	.23
Velocity	1	23	85.99	< .001	.79
Contrast	1	23	7.21	.013	.24
Diopter value	2	46	4.09	.023	.15
Velocity × contrast × TTC	1.74	40.11	5.54	.010	.19

*p*-values and degrees of freedom in the table incorporate Huynh–Feldt correction if appropriate. *df*_*Num*_ = numerator degrees of freedom. *df*_*Den*_ = denominator degrees of freedom. η_p_^2^ = partial eta-squared.

Our subjects’ TTC estimates were rather accurate in normal viewing, that is with full contrast and without blur (*M* = 0.08 s, *SD* = 0.37 s), which can be considered as a baseline. With regard to this baseline, reduced contrast led to *overestimation* of TTC (*M* = 0.11 s, *SD* = 0.26 s), but added dioptric blur *shortened* the TTC estimates (Fig. 2). The effect of dioptric blur was further investigated by two planned contrasts. The TTC estimates at 0 diopters were longer than the estimates of + 1.5 diopters, *F*(1, 23) = 5.62, *p* = 0.027, *η*_*p*_^2^ = 0.20, but the estimates at + 1.5 diopters did not differ significantly from those at + 3.0 diopters, *p* = 0.865.

**Figure 3:**
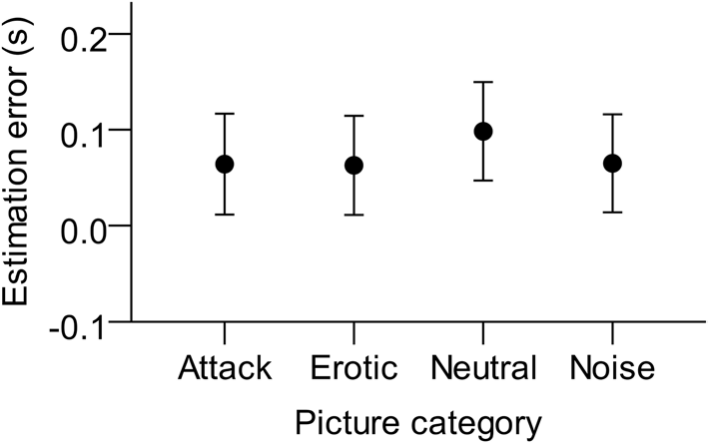
Mean TTC estimation errors (estimated TTC – actual TTC) as a function of picture category. Error bars represent ± 1 standard error of the mean across participants.

TTC was also overestimated when the images approached faster (*M* = 0.13 s, *SD* = 0.26 s), compared to the slow images (*M* = 0.01 s, *SD* = 0.25 s). Note that to keep TTC-values comparable, velocity was correlated with initial distance, which might explain this effect. Picture category had a significant effect on TTC estimates (see Fig. 3). We further explored this main effect with Bonferroni-corrected post-hoc tests, which resulted in significant differences for the comparisons attack vs. neutral (*t*(23) = 3.4, *p* = 0.013, *d*_z_ = 0.68), erotic vs. neutral (*t*(23) = 5.0, *p* < 0.001, *d*_z_ = 1.0), and noise vs. neutral (*t*(23) = 4.86, *p* < 0.001, *d*_z_ = 0.97). All other comparisons did not reach significance (*p* > 0.05). Approaching neutral images led to significantly larger TTC estimates than images of the other categories.

**Figure 4:**
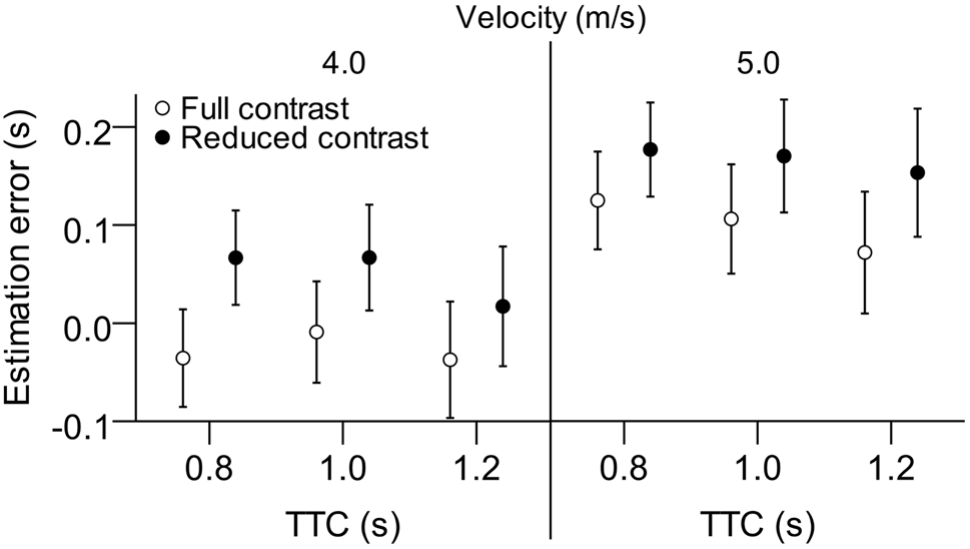
Mean TTC estimation errors (estimated TTC—actual TTC) as a function of velocity, simulated TTC and reduced contrast. Error bars represent ± 1 standard error of the mean across participants.

There was no significant main effect of TTC nor any interactions, with the exception of the threefold interaction of contrast, velocity, and TTC. Figure 4 reveals the nature of this threefold-interaction. The effect of reduced contrast to increase relative TTC overestimation at higher approach velocity was more pronounced with longer TTCs, and at lower approach velocity it was more pronounced with shorter TTCs.

#### Variable error (SD)

To investigate the uncertainty of the TTC estimations, we conducted a 4 (picture category) × 2 (velocity) × 3 (TTC) × 2 (contrast) × 3 (diopter value) repeated measures ANOVA on the variable error, that is the standard deviations of the TTC estimation error. Neither the main effect of picture category, nor any interaction including picture category reached significance, all *p* > 0.05. This allowed for aggregation across the factor picture category and for calculation of the variable error of the TTC estimates with 12 trials per experimental combination. We conducted a 2 (velocity) × 3 (TTC) × 2 (contrast) × 3 (diopter value) repeated measures ANOVA to examine effects on the variable error.

**Figure 5:**
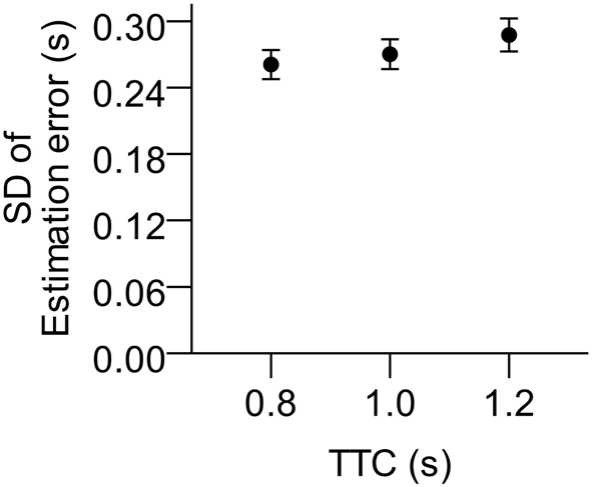
Mean standard deviation (*SD*) of TTC estimation errors (variable error) as a function of actual TTC. Error bars represent ± 1 standard error of the mean across participants.

Significant type 3 *F*-tests can be found in Table 2. The variable error increased significantly with TTC (see Fig. 5). Bonferroni-corrected post-hoc tests showed that the variation of TTC estimates was larger for an actual TTC of 1.2 s than for 0.8 s, *t*(23) = 3.57, *p* = 0.007, *d*_z_ = 0.73. Faster velocity tended to produce larger variable errors but failed to reach significance, *p* = 0.06.

**Table 2:** Significant main and interaction effects of type 3 *F*-tests on mean variable errors (*SD*).

	*df*_*Num*_	*df*_*Den*_	*F*	*p*	η_*p*_^*2*^
TTC	1.88	43.23	9.35	< .001	.29
Contrast × Velocity	1	23	10.01	.004	.30
Velocity × TTC	2	46	3.39	.04	.13

*p*-values and degrees of freedom in the table incorporate Huynh–Feldt correction if appropriate. *df*_*Num*_ = numerator degrees of freedom. *df*_*Den*_ = denominator degrees of freedom. η_*p*_^*2*^ = partial eta-squared.

**Figure 6:**
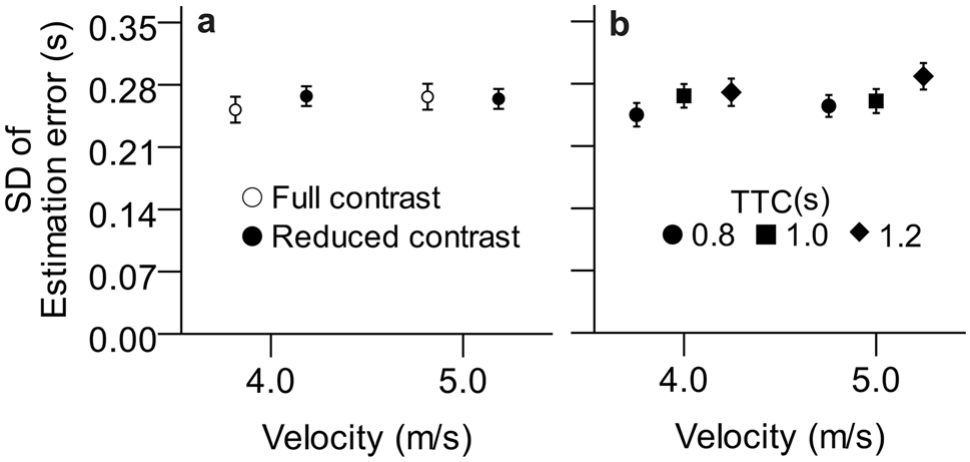
Mean standard deviations (*SD*) of TTC estimation errors (variable error) as a function of velocity, contrast condition (panel a), and actual TTC (panel b). Error bars represent ± 1 standard error of the mean across participants.

A significant interaction surfaced between contrast and velocity and between velocity and TTC (see Fig. 6). That is, slow targets at full contrast produced particularly low variable error. Fast targets with long TTC produced large variable error, whereas slow targets with short TTCs produced small variable error. Neither contrast nor diopter value, nor their interaction had a significant influence on the variable error of TTC estimations (all *p* > 0.50), except for the interaction of reduced contrast with velocity described above.

#### SAM ratings

**Figure 7:**
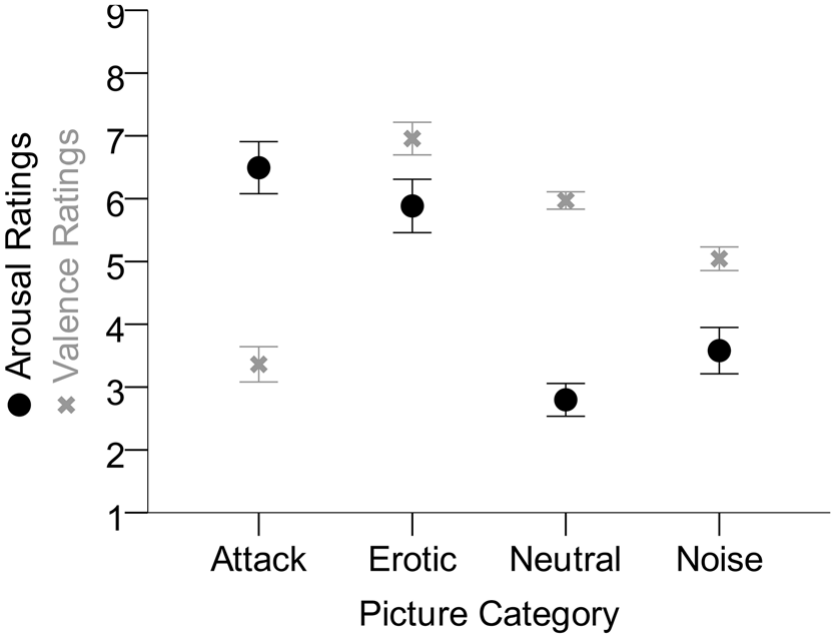
Mean arousal and valence ratings for the categories of pictures used as approaching objects. Error bars represent ± 1 standard error of the mean across participants.

SAM ratings are shown in Fig. 7. We calculated mean arousal and valence ratings for each picture category and analyzed those in two separate ANOVAs with picture category as 4-level repeated measures factor. The differences between the ratings for each picture category were significant, *F*_arousal_(2.40, 52.82) = 30.07, *p* < 0.001, *η*_*p*_^2^ = 0.58; *F*_valence_(2.33, 51.32) = 53.94, *p* < 0.001, *η*_*p*_^2^ = 0.71. For both dependent variables, we calculated Bonferroni-corrected post-hoc tests. All these tests were significant (*p* < 0.05), except for the comparison between attack and erotic images in the arousal ratings (*p* > 0.99). The neutral images received lower arousal ratings than all other picture categories. In contrast, the valence ratings were higher for neutral stimuli than for the noise and attack categories, but lower than for the erotic category. Remember that neutral pictures were associated with longer TTC estimates than the other pictures.

### Discussion

Other than hypothesized, contrast reduction and dioptric blur did not affect the variability of TTC judgements. As hypothesized, TTC was estimated longer for reduced contrast stimuli. This effect may be due to a slower perceived speed of stimuli with reduced contrast^18,20,35,60^. Alternatively, our finding may be explained with an increased perceived distance as a consequence of reduced contrast^21,22^. As the reduction of contrast impairs target-background separation, it would be akin to a crude heuristic of aerial perspective, which assigns distance to low-contrast objects and proximity to high-contrast objects^23^. Note that the explanations of slower perceived speed and larger perceived distance are not mutually exclusive. In fact, both explanations are interwoven as perceived slower speeds may amount to greater perceived distances in the prediction motion paradigm. Moreover, as our contrast reduction also increased the images’ luminance, the results do not allow to clearly disentangle the effects of contrast and luminance. We realize that previous findings^15–17^ indicated faster matching speed in high luminance. As higher matching speed is associated with lower perceived speed in high luminance, our results of longer estimated TTCs are compatible with these findings. Since the virtual semi-transparent mask decreased contrast and increased luminance, and led to longer TTC estimates, luminance appears not to be the only decisive factor in our task. Here, we outline that both high luminance and reduced contrast, as can be caused by cataracts, produced longer TTC estimates.

Without contrast reduction and dioptric blur, TTC was estimated rather accurately, indicating that overall participants processed the task correctly. In accordance with our hypothesis, TTC was estimated *shorter* when vision was blurred than when vision was clear. If TTC is judged exclusively on the basis of relative optical expansion, blur should not have affected TTC estimates. If, however, size estimates enter the equation, and dioptric blur has led to a bigger appearance of the stimuli, this could have resulted in a size-arrival effect^61,62^ leading to systematically shorter TTC estimates. However, blur circles of 0.02 m and 0.04 m at + 1.5 and + 3.0 diopters, respectively (diameter approximated^63^), lead to size differences of merely 2% to 6%, depending on diopter value and distance. These differences were rather small compared to the size differences typically necessary to demonstrate the size-arrival effect (e. g. 13% to 22%^62^). Moreover, considering that blur was induced using an optometrist’s trial frame—which altered not only the depicted stimuli, but the entire scene in the participants’ field of view—participants might have accelerated their responses because the blur manipulation was perceived as unpleasant or irritating. In this sense, the manipulation induced a much more immersive form of degradation, which in turn could have led to cautious behavior.

Moreover, neither arousal nor valence did moderate the relationship between visual degradation and TTC estimation. The largest effect was found for velocity. When images approached faster, TTC was estimated longer, in relative terms. This result is consistent with the convincing findings in other species, that rather than relative expansion rate, observers use simpler optical variables, or threshold values thereof, correlated with TTC to guide their estimates, such as relative velocity or size^27,64^. However, it is not the only interpretation considering the necessary confound that images which approached faster also started from farther away (due to the fully-crossed design) and were on average depicted smaller on the screen as they approached, the effect is quite plausible and again in accordance with a size-arrival effect. Taken together, this suggests that our participants also made use of a bigger-nearer heuristic to judge TTC^24–26^.

## Experiment 2: environmental stimulus degradation

The experiment described in this section was part of the second author’s academic thesis^47^. In this experiment, we investigated what effect ambient motion signals, as operationalized by simulated snowfall, have on TTC estimation. Given that the motion of an additional approaching object may shorten TTC estimates^65^, we expected to find shortened TTC estimates in the presence of snowfall if the snow moves in the same direction as the target stimulus (head wind). This would also be in line with the effects observed for simulated observer forward self-motion^5,44,45^. Accordingly, an opposite effect should occur in tail wind, which would simulate observer backward self-motion^44^. However, if observers discard the increased motion signals in the background, TTC estimates should remain unaffected. If they merely take snowfall of any kind as an indicator for caution, TTC estimates should be shorter in the presence of snow. Having found no interaction effect between visual degradation and the affective stimulus content in Experiment 1, we omitted the affective variable in Experiment 2.

### Method

#### Participants

21 students of psychology at the University of Mainz (15 women, 6 men; age: 19–38 years, *M* = 22.76 years, *SD* = 4.85 years) participated in the experiment for partial course credit. All had normal or corrected-to-normal visual acuity and had not participated in other TTC experiments before. The experimental procedures were in accordance with the principles of the Declaration of Helsinki, as described in Experiment 1^50^. Informed consent was obtained from each participant.

### Apparatus and stimuli

The participants sat in a darkened room, a chin rest stabilized their head position at 0.5 m viewing distance to the screen (52 × 29.4 cm, resolution 1920 × 1080 pixel). The stimuli were programmed in virtual space (Vizard 3.0), one virtual m corresponding to one real m, and presented with a DELL Precision 380 computer, graphics card NVIDIA Quadro FX3500, at a frame rate of 60 Hz.

**Figure 8:**
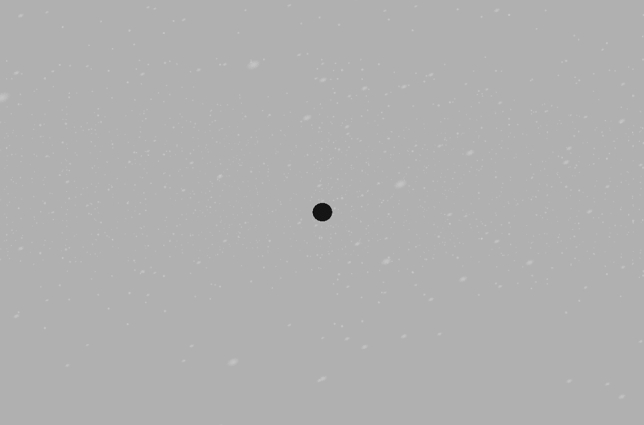
Example stimulus of Experiment 2, with snowfall.

The stimulus was a black sphere of 2 m width, frontally illuminated, in a uniform grey environment. In the snow conditions, moderate snowfall under different wind conditions was simulated using Vizard’s snow function and the Precipitation plug-in (the “ < object > .setSpeed” parameter was set to “2”). The snow was either falling straight downwards, or it was moderately blown towards the observer, away from the observer, or from right to left across the screen. Figure 8 shows an example stimulus with snowfall.

### Design and procedure

In a prediction motion paradigm, the sphere was depicted as approaching the participant at constant velocity and was blanked out after 1.5 s. The approaching sphere started at initial distances between 11 and 16 m and was occluded between 5 and 8.5 m from the observer. This corresponds to visual angles between 10.4º and 7.2º (initial) and between 22.6º and 13.4º (final). Participants had to extrapolate the motion of the sphere after it disappeared and to press a button when its front would have collided with them. TTC estimates were calculated as the time from the sphere’s disappearance to the participant’s button press. To discourage participants from basing their judgments on simple heuristics, we varied approach velocity and actual TTC (time from disappearance to collision with the front tip of the sphere).

The repeated-measures design consisted of three fully crossed factors: *snow condition* (5 levels: no snow, head wind (blowing frontally towards the viewer), tail wind (blowing away from the viewer towards the sphere), side wind and no wind), *velocity* (2 levels: 4 and 5 m/s), and *TTC* (3 levels: 1.0, 1.25 or 1.5 s). Because we did not want to create an oddball effect for the no-snow condition, we presented each factor combination twice for the four variations of the snow conditions (48 trials with snow) but four times for the no-snow condition (24 trials without snow). Thus, participants viewed 72 trials in total in randomized orders, starting each trial at their own pace. The experiment was interrupted for two short breaks to relax. Eight training trials with feedback (displaying the estimation error in ms and whether it was too short or too long), preceded the experiment, using the stimulus without snowfall. No feedback was given for the experimental trials.

### Results

#### Constant error

We computed TTC estimation errors (estimated TTC—actual TTC), averaged across the trials per factor combination, and analyzed those averages in a 5 (snow condition) × 2 (velocity) × 3 (TTC) ANOVA with repeated measures on all factors. We used an alpha level of 5% and report Huynh–Feldt corrections where appropriate. Significant effects on mean TTC estimation errors are shown in Table 3.

**Table 3:** Significant main and interaction effects of type 3 *F*-tests on mean constant errors.

	*df*_*Num*_	*df*_*Den*_	*F*	*p*	η_*p*_^*2*^
Snow	3.88	77.64	4.92	.002	.20
Velocity	1	20	57.85	< .001	.74
TTC	1.9	38.21	31.75	< .001	.61

*p*-values and degrees of freedom in the table incorporate Huynh–Feldt correction if appropriate. *df*_*Num*_ = numerator degrees of freedom. *df*_*Den*_ = denominator degrees of freedom. η_*p*_^*2*^ = partial eta-squared.

**Figure 9:**
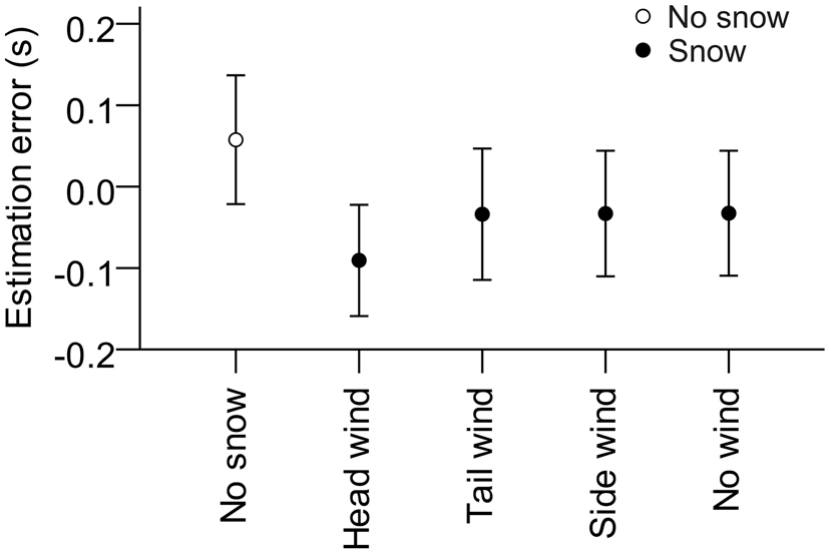
Mean TTC estimation errors (estimated TTC—actual TTC) as a function of snow condition. Error bars represent ± 1 standard error of the mean across participants.

We found a significant effect of snow and calculated two separate contrasts. Firstly, we compared the pooled means of the four ‘snow’ conditions to the ‘no-snow’ condition. Corresponding to our hypothesis, snow produced a significant shortening of TTC estimates, *F*(1, 20) = 11.95, *p* = 0.002, *η*_*p*_^2^ = 0.374. On average, TTC was overestimated by 0.058 s (*SD* = 0.488 s) without snow and underestimated by 0.048 s (*SD* = 0.469 s) in the presence of virtual snowfall (see Fig. 9). Secondly, we compared the effect of head wind against tail wind. They did not differ significantly, *p* = 0.186.

**Figure 10:**
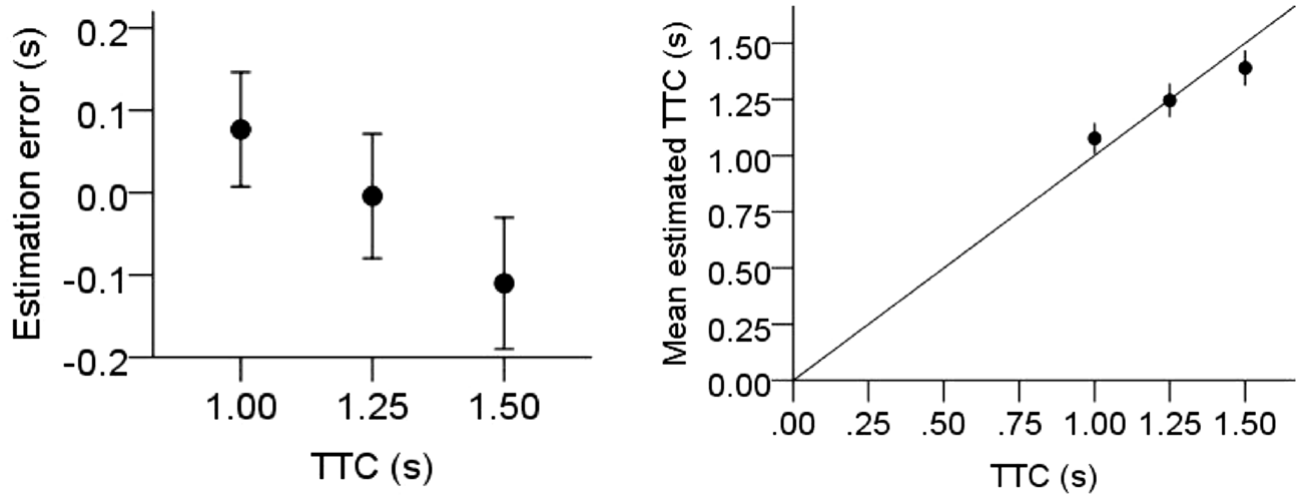
Left: Mean TTC estimation errors (estimated TTC—actual TTC) as a function of actual TTC. Right: TTC estimates as a function of actual TTC. Error bars represent ± 1 standard error of the mean across participants.

Velocity and actual TTC had the usual effects. TTC was overestimated for fast object approaches (*M* = 0.08 s, *SD* = 0.48 s), compared to slow object approaches (*M* = – 0.10 s, *SD* = 0.46 s). Moreover, the average TTC estimation error changed from overestimation for the shortest TTC to underestimation for the longest actual TTC (see Fig. 10). There were no significant interactions, all *p* > 0.09.

#### Variable error (SD)

To examine the uncertainty of the TTC estimations, we conducted a 5 (snow condition) × 2 (velocity) × 3 (TTC) repeated measures ANOVA on the variable error, that is the standard deviations of the TTC estimation error. The results revealed no significant effects for any of the factors or their interactions, all *p* > 0.05.

### Discussion

Simulated snowfall shortened TTC estimates of a frontally approaching object. This effect was independent of the direction of the snow. Thus, the presence of snow produced a general effect of underestimating TTC, which cannot be attributed to the direction of the snow’s motion. In this case, the effect should have varied according to the direction of the snowflakes’ motion. For instance, head wind should have caused TTC underestimation if it were suggestive of self-motion toward the target, as had been the case in other studies when optic flow did specify self-motion^5,44,45^.

What could have produced the observed TTC underestimation? Possibly, the snow was perceived as distracting or dangerous regardless of its specific direction of motion. It has been previously observed that the presence of task-irrelevant background objects can shorten TTC estimates^65^. In this regard, it is important to highlight a detail of our experimental set-up. The snow in our experiment was rather fine-grained and did not occlude the target at any point of time (see Fig. 8). Instead, the target obscured the snow whenever they overlapped. Therefore, the snow might have been grouped with the scene’s background, which seems reasonable as our simple scene did not provide any other target-external depth cues. The “background” information derived from the occluded snow might thus have been factored in the TTC estimation and moved the target closer to the observer. Similar effects have been found in distance estimation^66^. Additionally, the background snow could have affected the decoding of the object’s speed, leading to speed overestimation and consequently shorter estimated TTC. Similar behavior has been observed with respect to random visual background noise^67,68^. As the snowfall presented here was only visible in the background, this could have resulted in a similar misperception of target speed during the visible approach.

In conclusion, since we did not find the slightest trend towards a *lengthening* of TTC estimates in the presence of snowfall, not even with added tail wind, a hazardous influence of snowfall on TTC estimation in terms of increasing overestimations is unlikely. Note, however, that we have neither simulated real snowfall nor real driving, such that our finding should be seen as a reasonable prediction rather than a conclusive fact with regard to actual driving.

## General discussion

We have introduced observer-related and environmental stimulus degradations to a classical TTC estimation task. In Experiment 1, we found that reduced stimulus contrast lengthened TTC estimates of a frontally approaching object, whereas dioptric blur shortened estimates. Both were effects that cannot be attributed to increased variability of TTC estimation. Neither reduced contrast nor dioptric blur changed the estimations’ uncertainty.

The first finding is in accordance with the effect of reduced contrast on TTC estimation for fronto-parallel stimuli^35^. We consider the slowing of perceived speed under reduced contrast^18,20,60^ or an equivalent increase in perceived distance^22^ as the most probable explanation. However, as the mask we used to reduce contrast at the same time increased luminance, an alternative interpretation is possible. The findings obtained for frontoparallel stimuli that brighter objects appear to move slower^15–17^, might be applicable to the sagittally approaching stimuli we have used here. In this case, luminance increase might explain the effect, but such a luminance effect has been absent in our previous studies^34^.

The interpretation that lower contrast increased perceived distance is consistent with the lowered collision detection sensitivity in the presence of fog^33^. It is likewise consistent with the perception of increased distance in fog. Note, however, that the semi-transparent mask we used is not directly comparable to fog, since it remained the same intensity at all distances. Under foggy conditions in the real world, more distant objects are harder to detect than objects closer to the observer. The visual impairment introduced in Experiment 1 may rather be comparable to those caused by cataracts. Simulated cataracts have been found to slow detection of hazards^69^. Alternatively, or additionally, reduced contrast and higher luminance could have caused distance overestimation akin to aerial perspective. Be this as it may, they were associated with an overestimation of TTC, which is potentially dangerous in cases where the approaching object should be avoided, as would be the case in many traffic situations.

In contrast, dioptric blur as induced by the optician’s lenses could easily be recognized as visual degradation and consequently presented a more immersive degradation. This might have prompted cautious behavior because of the perceived deficiencies, possibly combined with a size-arrival effect due to the larger retinal image produced by the blurred contours. Thus, the observers may have adjusted their judgments towards a shorter TTC in reaction to the stimulus degradation. In a driving context, blurry vision could likewise trigger a safety strategy resulting in larger safety margins.

The arousal induced by our stimulus content also influenced TTC estimates. TTC for neutral images was estimated longer than TTC for all other conditions. This effect might result from the higher arousal induced by images involving attack, erotic stimuli, or noise (see Fig. 7), which is in accordance with an arousal advantage effect^52,53^. A discussion of potential mechanisms underlying this effect is provided in one of our previous experiments^53^. However, the effect of arousing stimulus content did not affect the influence of reduced contrast or dioptric blur on TTC estimation.

In Experiment 2, we found shorter TTC estimates when we added simulated snowfall, changing from slight overestimation without snow to slight underestimation with falling snowflakes. How can we explain this effect? As outlined above, the snowfall provided occlusion information in an otherwise sparse environment. The snow was occluded whenever it overlapped with the target, providing depth cues not present in scenes without snow. If these depth cues were taken into account, the snow might have been grouped to the background of the scene and the object thus appeared to be closer to the observer.

An alternate explanation can be derived from the effect of local motion transients on TTC estimation. Previous studies have observed dynamic background noise to modulate TTC estimates and proposed that the noise could lead to speed overestimation of visible and occluded objects^67,68^. Accordingly, shorter TTC estimates were found in the presence of dynamic background noise, similar to the effect in Experiment 2. The snow we simulated was smooth and continuous, but might still have been classified as noise by the visual system, regardless of the wind direction. This could have led to an overestimation of the visible object’s speed, which would be in agreement with the results of Battaglini and colleagues.

In contrast, the reduction of the target-background separation caused by the semi-transparent mask did not provide additional depth information, but made the target more difficult to recognize, which might have induced the false impression of enlarged distance or decreased speed, as described earlier^18,22,23,60^. Taken together, the effect of vision degradation on TTC estimation seems to depend on the specific form of degradation. Dioptric blur is often found as observer-based degradation and could trigger precautionary behavior, which could result in a safety strategy in meaningful contexts, such as driving. However, if vision is impaired due to falling snow or reduced contrast, the degradation effect on TTC estimation seems to depend more strongly on how it changes the information available in the visual scene. This is indeed compatible with previous findings from speed perception^20^. Here, participants perceived speeds as faster in fog obscuring more distant objects. With uniform contrast or an artificial anti-fog condition obscuring closer objects, however, speeds were perceived as slower than actual.

Some shortcomings should be addressed that potentially limit the explanatory power or generalizability of our results. We consciously employed a simple prediction motion task with driving-unrelated stimuli in our experiments. This was done to disentangle the basic effect of vision degradation on TTC estimation from other potentially moderating factors, such as arousal, and from more complex effects on driving behavior. Consequently, no precise predictions can be derived on how visual degradation would affect driving behavior. However, we hypothesize that the precautious behavior observed in the observer-internal degradation should be even more prominent in meaningful and potentially risky driving situations. Additionally, the contrast reduction employed in this experiment was different from the appearance of fog^20^ and more similar to symptoms associated with cataracts.

However, symptoms of cataract are also more complex, as they also include dioptric blur and color fading. In this regard, it would be interesting to test participants with actual internal impairments (resulting from cataract or glaucoma) in TTC estimation. As outlined in the introduction, several studies have measured general driving performance of cataract or glaucoma patients^13,70^, but to our knowledge, TTC estimation experiments with visually impaired participants wait to be conducted. Finally, since no condition of static snow was included in Experiment 2, the effect of snowfall on TTC estimation might have been produced either by the occlusion or by the distraction associated with the task-irrelevant motion in the scene. Nevertheless, we assume that occlusion information shortens TTC estimates as different motion directions have affected TTC estimation in a similar way.

In conclusion, stimulus degradation has adverse effects on TTC estimation performance. Reduced contrast accompanied by higher luminance of the stimulus lengthened the estimates, potentially increasing the risk of accidents. This could be related to a false impression of decreased speed or increased distance, as a result of a reduced target-background separation. In contrast, dioptric blur and snowfall led to shorter TTC estimates. Regarding dioptric blur, this could be interpreted as increased caution due to perceived internal deficiencies, which could trigger a safety strategy in more meaningful contexts. Our findings underscore the importance to investigate how specific visual impairments may affect the cues available for TTC estimation. If the degradation of visual cues is internal and observer-related, a safety mechanism could be prompted resulting in cautious behavior and thus shorter TTC estimates.

## Data Availability

The datasets generated and/or analyzed during the current study are available in the Gutenberg Open repository, http://doi.org/10.25358/openscience-5669.
